# Impact of a Multimodal Antimicrobial Stewardship Program on *Pseudomonas aeruginosa* Susceptibility and Antimicrobial Use in the Intensive Care Unit Setting

**DOI:** 10.1155/2011/416426

**Published:** 2011-05-19

**Authors:** Douglas Slain, Arif R. Sarwari, Karen O. Petros, Richard L. McKnight, Renee B. Sager, Charles J. Mullett, Alison Wilson, John G. Thomas, Kathryn Moffett, H. Carlton Palmer, Harakh V. Dedhia

**Affiliations:** ^1^Division of Infectious Diseases, West Virginia University (WVU), Morgantown, WV 26506-9163, USA; ^2^School of Pharmacy, West Virginia University (WVU), Morgantown, WV 26506-9520, USA; ^3^Pharmacy Services, WVU Hospitals, Morgantown, WV 26506-8045, USA; ^4^Division of Pediatrics, West Virginia University (WVU), Morgantown, WV 26506-9214, USA; ^5^Division of Surgery, West Virginia University (WVU), Morgantown, WV 26506-9238, USA; ^6^Department of Pathology, West Virginia University (WVU), Morgantown, WV 26506-9203, USA; ^7^Department of Internal Medicine, West Virginia University (WVU), Morgantown, WV 26506-9160, USA; ^8^Division of Pulmonary-Critical Care Medicine, West Virginia University (WVU), Morgantown, WV 26506-9166, USA

## Abstract

*Objective*. To study the impact of our multimodal antibiotic stewardship program on *Pseudomonas aeruginosa* susceptibility and antibiotic use in the intensive care unit (ICU) setting. *Methods*. Our stewardship program employed the key tenants of published antimicrobial stewardship guidelines. These included prospective audits with intervention and feedback, formulary restriction with preauthorization, educational conferences, guidelines for use, antimicrobial cycling, and de-escalation of therapy. ICU antibiotic use was measured and expressed as defined daily doses (DDD) per 1,000 patient-days. *Results*. Certain temporal relationships between antibiotic use and ICU resistance patterns appeared to be affected by our antibiotic stewardship program. In particular, the ICU use of intravenous ciprofloxacin and ceftazidime declined from 148 and 62.5 DDD/1,000 patient-days to 40.0 and 24.5, respectively, during 2004 to 2007. An increase in the use of these agents and resistance to these agents was witnessed during 2008–2010. Despite variability in antibiotic usage from the stewardship efforts, we were overall unable to show statistical relationships with *P. aeruginosa* resistance rate. *Conclusion*. Antibiotic resistance in the ICU setting is complex. Multimodal stewardship efforts attempt to prevent resistance, but such programs clearly have their limits.

## 1. Introduction

Intensive care units (ICUs) have the highest density of antimicrobial use and the highest rates of bacterial resistance. Institution-wide and specific unit-based antibiotic stewardship initiatives have been advocated by many as a way to preserve the utility of antimicrobial agents [1]. The Infectious Diseases Society of America (IDSA) and the Society for Healthcare Epidemiology of America (SHEA) have developed guidelines for antibiotic stewardship to help combat bacterial resistance [2]. The two core tenets of these guidelines are use of “*prospective audits with intervention and feedback”* and “*formulary restriction and preauthorization.”* Additional elements of an antibiotic stewardship program could include: *education*, *guidelines and clinical pathways*, *antimicrobial cycling*, and* de-escalation of therapy*. 

Prior to the release of the IDSA/SHEA guidelines, a multimodal institution-wide antimicrobial stewardship program was developed at our university hospital which incorporated many of the elements listed in the guideline. Adult ICUs were especially targeted by our stewardship initiatives due to the high baseline use of antibiotics. A major impetus for developing our program was to improve or preserve* Pseudomonas aeruginosa* susceptibility given the limited number of antibiotics with activity against this important pathogen. In particular, we had witnessed a jump in *P. aeruginosa* resistance to ciprofloxacin from 38% in 2003 to 56% in 2004 in the adult ICUs before the start of the program. The objectives of this study are to describe how implementation of an institutional multimodal stewardship program affected *P. aeruginosa* susceptibility and antibiotic use in the ICU setting.

## 2. Material and Methods

### 2.1. Hospital and Stewardship Efforts

Our institution is a 531-bed academic medical center. Adult ICUs include a Cardiothoracic Unit, Coronary Care Unit, closed-model Medical Intensive Care Unit (MICU), and a Surgical Intensive Care Unit (SICU). Both the MICU and SICU have dedicated unit-based critical care physicians and clinical pharmacy specialists. In addition, clinical staffing pharmacists verify orders on all shifts and are instructed to intervene on restricted medication orders if used outside of criteria. They also make sure that the teams are following the antibiotic cycling. 

During the study period, no significant changes were implemented in terms of infection control practices except for an increase in alcohol-based hand sanitizer use during the 2009-2010 H1N1 influenza pandemic. Wall dispensers were placed at the entrance to each hospital patient's room and at all ICU entrances. This study was conducted in accordance with the Declaration of Helsinki (1964) and was granted exempt status by the University Institutional Review Board.

Our stewardship program incorporates prospective audits with intervention and feedback, formulary restriction and preauthorization, educational noon conferences for physicians and pharmacists, institutional pocket card guidelines, and a ventilator-associated pneumonia (VAP) antimicrobial cycling protocol with streamlining/de-escalation. The stewardship program was implemented in step-wise fashion starting in the fall of November 2004 with the development of a new high-peak aminoglycoside dosing protocol, and antibiotic educational initiatives including conferences. In January of 2005, criteria-based restrictions were placed on broad spectrum antibacterials. In particular, the new criteria restricted all fluoroquinolone use as first-line agents in the ICU. Ciprofloxacin, the ICU fluoroquinolone of choice, was deemed appropriate only in the setting of presumed or documented infections with *Pseudomonas aeruginosa *in patients allergic or intolerant to *β*-lactams or aminoglycosides. In July 2005, the VAP protocol was initiated as a way to increase the heterogeneity of antibiotic use. The antibiotic selections were broadly based on guidelines published by the American Thoracic Society [3]. Cycling of gram-negative agents (a carbapenem, cefepime, and piperacillin-tazobactam) was performed quarterly whereas cycling of gram-positive agents (vancomycin and linezolid) was performed semiannually. The VAP protocol was also designed to reduce the amount of ciprofloxacin and ceftazidime use given the increased resistance rates at the time. Imipenem was the primary carbapenem used during 2003–2009. A formulary switch to doripenem was made for 2010. Limited use of meropenem was permitted throughout the study period. De-escalation involved a switch to a narrower spectrum agent of the same antibiotic class (imipenem to ertapenem, cefepime to ceftriaxone, and piperacillin-tazobactam to ampicillin-sulbactam) at 48–72 hours. De-escalation also involved the discontinuation of the empiric gram-positive agent if MRSA was not isolated. Ceftazidime use was discouraged in favor of cefepime throughout the ICU setting. Finally, in August of 2005, pocket cards with antibiograms and institutional restriction and usage guidelines were distributed to physicians and pharmacists along with educational conferences. This has since become an annual event for incoming house staff. In addition, the pocket card data is available on the institution's intranet website. 

Since it was not logistically possible to assess each segment of this multimodal program on antimicrobial resistance in the ICU setting, we decided to focus our assessment on the global use of anti-pseudomonal agents in the adult ICUs and compared the use of these agents with *P. aeruginosa* resistance in the same ICUs.

### 2.2. ICU Antibiotic Utilization Data

ICU antibiotic usage was collected from patient billing databases for 2003 to 2008. Billing data is derived from nursing medication administration data. Antibiotic usage was converted from total grams used to defined daily doses (DDD) per 1,000 patient-days to make year-to-year comparisons. The DDD conversion factors were developed from the World Health Organization definitions for individual antibiotics (http://www.whocc.no/atc_ddd_index/). This is one of the most commonly used methods to measure antibiotic consumption [4, 5].

### 2.3. ICU Resistance Determination

Nonduplicative adult ICU specimens from all sources were analyzed in this study. Susceptibility was determined using Vitek and Vitek 2 automated systems (bioMérieux). Carbapenem resistance rates were represented by imipenem resistance in this study since it was the carbapenem agent used the most and since doripenem susceptibility testing was not available with our automated system during the majority of the study. Automated susceptibility testing for piperacillin-tazobactam and cefepime were not performed until 2005 when these agents became formulary agents. Susceptibility results were interpreted according to Clinical Laboratory Standards Institute (CLSI) guidelines. Resistance data was expressed and compared using CLSI “resistant” breakpoints rather than “nonsusceptible” breakpoints. Breakpoint values did not change during this study. Clinical laboratory data were exported to The Surveillance Network (TSN) Database (Focus Technologies, Herndon, VA) for benchmarking. Semi-annual reports were obtained from TSN for analysis.

### 2.4. Statistical Analysis

Linear correlation analysis was performed to assess possible relationships between antibiotic usage and resistance in *P. aeruginosa*. All data was assessed using JMP V9.0 Statistical software (SAS Institute, Inc, Cary NC.). Univariate analysis of the use and resistance rates of the same antibiotic was performed followed by bivariate analysis looking at possible relationships with other antibiotics. *P*-values of <.05 were considered statistically significant.

## 3. Results

One of the most compelling findings of our study is that overall antibiotic use decreased in the ICU setting as well as in the whole institution between 2004 and 2007. ICU use of key anti-pseudomonal agents decreased from 412 DDD/1,000 patient-days in 2004 to 346 DDD/1,000 patient-days in 2007. The proportion of antibiotics in the overall pharmacy medication budget decreased from 15.8% in 2003 to 8.3% in 2007. Antibiotic costs decreased as some agents became generic and overall pharmacy purchase of some expensive biological and cancer agents also increased, thus the antibiotic proportion of the pharmacy budget decreased. Unfortunately, in 2010, the ICU use of anti-pseudomonal agents increased to 456 DDD/1,000 patient-days. This most likely reflected the increased incidence in resistant organisms reported in 2008–2010. Changes in ICU anti-pseudomonal usage patterns between 2003 and 2010 appear to reflect a greater degree of heterogeneity. The proportion of antibiotics used in highest frequency at the beginning of the study period decreased, and the proportion of less frequently used antibiotics increased by the end of the study period. Usage patterns of anti-pseudomonal agents in the adult ICUs are reported in Table 1. Fluctuation in usage appears to be affected by the VAP protocol. Since only three gram-negative agents were used in the VAP protocol on a quarterly cycle, this required that the first and fourth quarters would use the same agent within the year. 

**Figure 1:**
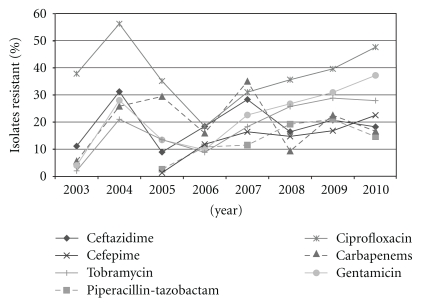
Percent of intensive care unit *P. aeruginosa* isolates resistant to various anti-pseudomonal agents.

Yearly ICU antibiotic resistance in *Pseudomonas aeruginosa* appears in Figure 1. The most significant change in antimicrobial resistance patterns was observed in the reduction of ciprofloxacin-resistant *P. aeruginosa* from 56.2% in 2004 to 18.4% in 2006. Ceftazidime use decreased throughout the study period. Ciprofloxacin use decreased substantially until 2007 while cefepime, tobramycin, and piperacillin-tazobactam increased throughout the study. Unfortunately, the ciprofloxacin resistance rate increased to 31.0% in 2007 and then to 47.6% in 2010. This rebound of resistance appears to be consistent with increased use of ciprofloxacin during 2008–2010. However, the resistance to ciprofloxacin was still lower than the preprogram rates. Ceftazidime-resistant *P. aeruginosa* also decreased from 31.2% in 2004 to 18.4% in 2006. Unfortunately, the resistance rate increased to between 16.4% and 20.7% since 2007. Piperacillin-tazobactam and cefepime were not added to the hospital formulary until the later part of 2003, so their susceptibilities were not routinely performed until 2005. Resistance to these two agents appears to have increased as their use has increased since 2005. In general, the resistance rates do not appear to be impacted by any lapses in standard infection control practices as clonal resistance outbreaks were not reported during the study period.

Data from the linear correlation analysis are reported in Table 2. In univariate analysis of each antibiotic's usage and resistance, statistical significance was not reached despite the fact that 35% of the variability in ciprofloxacin resistance and 54% of variability in cefepime resistance appeared to be associated with the variability in usage of those agents. In bivariate analysis, tobramycin use was inversely correlated with ciprofloxacin resistance and cefepime use was significantly associated with ceftazidime resistance.

## 4. Discussion

The design of this practice-based study was not to specifically measure the impact of any individual initiative with changes in resistance patterns, but rather to look at the impact of a multimodal stewardship program on ICU Pseudomonas susceptibility. A majority of published studies on antibiotic use and resistance have reported increased resistance with increased use of antibiotics [6]. Our study is one in the minority of studies to show a decrease in ciprofloxacin and ceftazidime resistance among ICU *P. aeruginosa* isolates with decreased ciprofloxacin and ceftazidime use. Nguyen and colleagues also recently reported a decrease in ciprofloxacin-resistant *P. aeruginosa* in a case-control study; however, their analysis was not limited to the ICU setting [7]. Of course, establishing a direct link between antibiotic use and any degree of attributable resistance is not easily done. 

Enforcement of class restrictions can reduce the use of particular antibiotics. A common consequence is that increases in other classes often result in increased resistance to those agents. Such relationships have been referred to as “balloon squeezing” resistance patterns [8]. Antibiotic restrictions at our hospital along with the VAP cycling protocol appeared to affect overall usage patterns in the ICUs. The VAP cycling protocol was specifically designed to improve susceptibility to ciprofloxacin and ceftazidime, as neither was included in the cycling protocol. Cefepime has replaced ceftazidime at many hospitals, but it may not protect against cephalosporin resistance in Pseudomonas. Not only did we find a trend in the univariate assessment of cefepime use and resistance, but we did see an association with cefepime use and ceftazidime resistance. 

We cannot easily explain the increase in *P. aeruginosa* ciprofloxacin resistance in 2007–2010 after the initial reduction in ciprofloxacin usage. However, we did see an increase in the use of ciprofloxacin usage in our ICUs during the first half of 2007 and in 2010. This may explain the *r^2^* value of 0.35. Interestingly, we also witnessed an increase in multiple-class resistance in 2007 and 2010. This may be a reflection of national trends and perhaps a consequence of upregulation of multidrug efflux pumps with use of other agents [9–11]. At least one other group of investigators has postulated the maintenance of multidrug-resistant* P. aeruginosa *during an antibiotic cycling study [12]. 

The major limitation to our study is the inability to separate out the consequences of each initiative in our multimodal program. In addition, we could not assess any contribution to resistance from horizontal transmission due to lapses in infection control, or as a result of antibiotic use outside of the ICU or in the community. In addition, much smaller amounts of non-pseudomonal antibiotics were used in the ICUs during the time of study and not factored into our analysis. Despite these limitations, we were still able to highlight a few trends by the temporal relationships of the antibiotic usage patterns and the resistance patterns. Our data also suggests that despite interventions such as the VAP protocol and limiting ciprofloxacin and ceftazidime use, it is very difficult to reduce anti-pseudomonal resistance in the ICU setting.

## 5. Conclusions

We are one of the earlier institutions to provide results from an antibiotic stewardship program that uses multiple measures advocated in the IDSA/SHEA guidelines to prevent or delay ICU antimicrobial resistance. Our multimodal program appears to be associated with certain beneficial trends in antibiotic usage and resistance. However, these efforts may not always be able to control natural resistance patterns. More research is required in this area.

## Figures and Tables

**Table 1:** Intensive care unit anti-pseudomonal antibiotic utilization (2003–2010).

	2003	2004	2005	2006	2007	2008	2009	2010
Cefepime	4.5	4.0	18.5	36.5	68.5^†^	**92.5**	36.0	**90.5** ^†^
Ceftazidime	**90.5**	**62.5**	53.0	38.0	24.5	39.0	23.5	25.5
Piperacillin-tazobactam	87.0	124.5	127.5	115.5^†^	97.5	**139.0**	130.0^†^	**133.0**
Carbapenems	30.0	52.5	53.0	60.0	48.5	**112.** ^†^	**88.5 **	78.5
Ciprofloxacin	**192.5**	**148.0**	57.8	25.5	40.0	66.5	48.0	87.0
Gentamicin	10.5	20.5	**40.0**	**26.5**	8.5	17.5	10.5	11.0
Tobramycin	1.0	0.5	42.5	**56.5**	**58.0**	48.0	35.0	31.0

Data expressed as defined daily dose (DDD) per 1000 patient-days.

The two years with the highest percentage of specific antibiotic use are bolded.

^†^Year with two cycles of this antibiotic in VAP protocol.

**Table 2:** Assessment of correlation between *P. aeruginosa* resistance and antibiotic usage.

*Parameters*	Correlation coefficient (*r*)	*r* ^2^	*P *value
*Univariate assessments*			
Ciprofloxacin	0.593	0.351	.121
Ceftazidime	0.338	0.114	.413
Cefepime	0.734	0.538	.097
Piperacillin-tazobactam	0.386	0.149	.450
Carbapenems	0.219	0.048	.602
Gentamicin	0.374	0.139	.362
Tobramycin	0.184	0.034	.664

*Bivariate assessments* ^†^			
Ciprofloxacin resistance	−0.735	−0.540	.038
Tobramycin usage			
Ceftazidime resistance	0.966	0.934	.002
Cefepime use			

^†^Only relationships with *P* values <.05 are listed.
